# Infective endocarditis with Osler’s nodule in a patient with Osler’s disease: a case report and review of the literature

**DOI:** 10.1186/s13256-022-03427-2

**Published:** 2022-05-29

**Authors:** Genki Naruse, Takatomo Watanabe, Hiroyuki Okura

**Affiliations:** 1 Department of Cardiology, Gifu University Graduate School of Medicine, 1-1 Yanagido, Gifu, 501-1194 Japan; 2 Division of Clinical Laboratory, Gifu University Hospital, Gifu, Japan

**Keywords:** Extracerebral infections, Hereditary hemorrhagic telangiectasia, Infective endocarditis, Osler–Weber–Rendu disease, Skin lesions, Case report

## Abstract

**Background:**

Hereditary hemorrhagic telangiectasia, also known as Osler–Weber–Rendu disease, induces arteriovenous malformations in visceral organs. Arteriovenous malformations increase the risk of severe infections and are a common complication associated with hemorrhagic telangiectasia. However, cases of endocarditis associated with hemorrhagic telangiectasia are rarely reported. Although hemorrhagic telangiectasia causes erythematous macules on the extremities, these macules are usually painless. We encountered a rare case of infective endocarditis in a patient with Osler–Weber–Rendu disease.

**Case presentation:**

A 52-year-old Japanese woman who was diagnosed with hemorrhagic telangiectasia 5 years prior presented to our hospital with fever and muscular pain. She had erythematous nodules and tenderness on the finger, heel, and toe, suggestive of Osler’s nodes. A physical examination revealed tachycardia with a 3/6 pansystolic murmur. A transesophageal echocardiogram showed vegetations along the atrial side of the mitral valve and mild mitral regurgitation because of prolapse of the anterior commissure. Methicillin-sensitive *Staphylococcus aureus* was identified in the blood cultures. Detection of distinctive skin lesions, so-called Osler’s nodes, was the symptomatic key to early diagnosis, and the patient was treated without surgery. She was discharged with negative blood cultures after a 6-week intravenous antibiotic administration.

**Conclusions:**

Our report highlights the importance of considering the risk of extracerebral infections including endocarditis in hemorrhagic telangiectasia. This rare case effectively demonstrates the importance of proper diagnosis of skin lesions.

## Background

Hereditary hemorrhagic telangiectasia (HHT) or Osler–Weber–Rendu disease (Osler’s disease) is an autosomal dominant disorder characterized by vascular anomalies that may visually develop in many organs [1]. Two main types of HHT represent 80% of cases: HHT type 1 results from mutations in the encoding for the endoglin (*ENG*) gene, while HHT type 2 results from mutations in the activin type II-like receptor kinase 1 (*ACVRL1*) gene (encoding for the activin receptor-like kinase). The *ENG* and *ACVRL1* genes code for proteins of the TGF-beta receptor, which is involved in proper blood vessel development [2]. Four main clinical diagnostic criteria have been established and carefully delineated for HHT: epistaxis, telangiectasia, visceral lesions, and an appropriate family history [3]. Diagnosis of HHT is confirmed when any three of the given criteria are presented.

Vascular anomalies in visceral organs include cerebral, spinal, hepatic, pancreatic, and pulmonary arteriovenous malformations (AVMs). These AVMs allow for direct communication between pulmonary and systemic circulation and expose patients with HHT to increased risk of severe infections. Although numerous cases of severe infections, such as cerebral abscesses, septicemia, arthritis, and osteomyelitis, have been reported, cases of endocarditis associated with HHT are relatively rare [4–7]. Here, we present a case of infective endocarditis in a patient with HHT.

## Case presentation

**Fig. 1:**
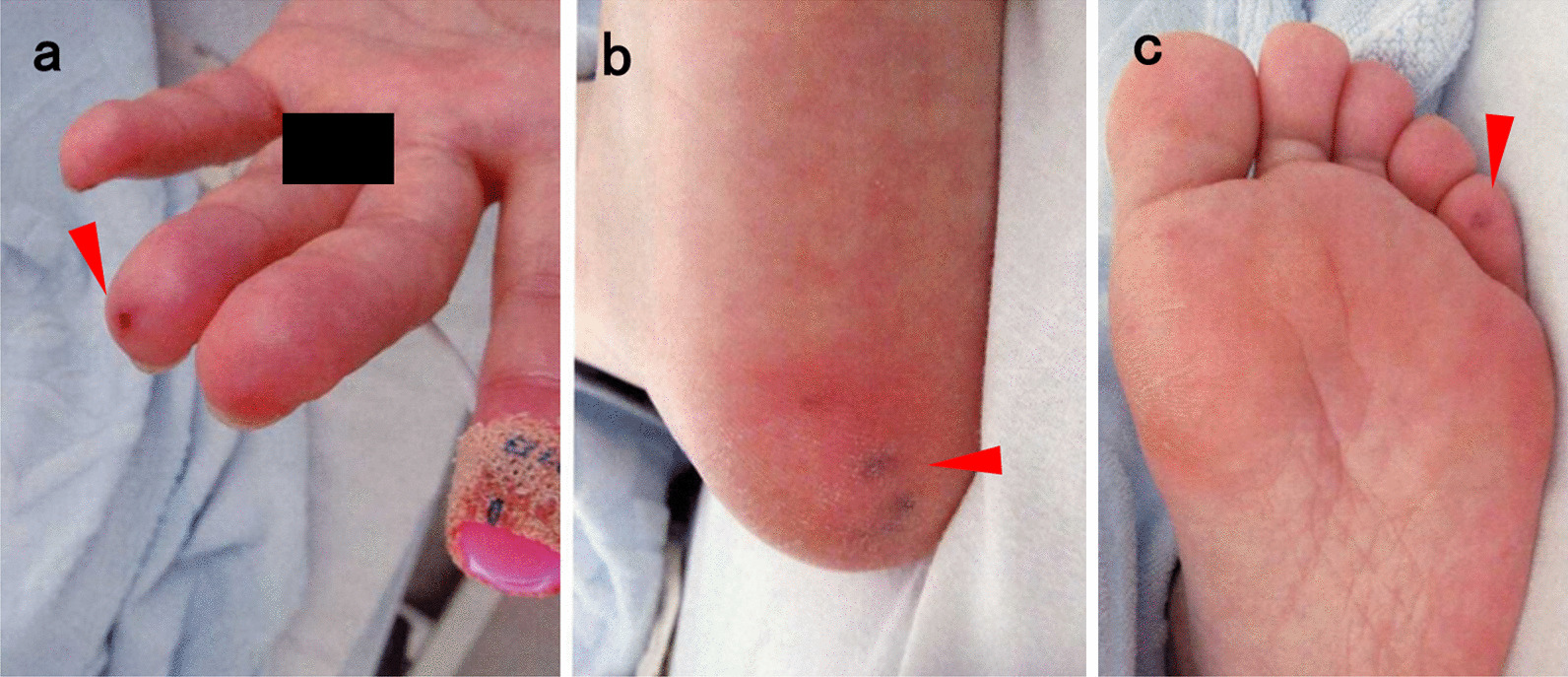
Osler’s node (arrowhead) on the right ring finger (**a**), right heel (**b**), and fifth toe (**c**)

The patient was a 52-year-old Japanese woman with history of recurrent nasal bleeding who was diagnosed with Osler’s disease 5 years prior. She complained of fever and muscular pain but had no recent history of tooth pain or dental procedure. She also denied any history of illicit drug use. Initial vital signs revealed a blood pressure of 114/65 mmHg, pulse rate of 112 beats/minute, temperature of 37.6 °C, respiratory rate of 22 breaths/minute, and oxygen saturation of 98% in room air. Physical examination revealed tachycardia with a 3/6 pansystolic murmur. Skin examination revealed erythematous tender nodules on the right ring finger, right heel, and fifth toe, suggestive of Osler’s nodes (Fig. 1). There was no evidence of splinter hemorrhages, conjunctival hemorrhages, or Janeway lesions. Laboratory examination showed that the hemoglobin level was 10.8 g/dL (11.6–14.8 g/dL), and the red blood cell count was 371 × 10^4^/µL (386–492 × 10^4^/µL). The leukocyte count, platelet count, and C -reactive protein level were 13.4 × 10^3^/µL (3.3–8.6 × 10^3^/µL), 4.1 × 10^4^/µL (15.8–34.8 × 10^4^/µL), and 11.5 mg/dL (< 0.14 mg/dL), respectively. The serum creatinine, procalcitonin, and brain natriuretic peptide levels were 2.17 mg/dL (0.46–0.79 mg/dL), 4.85 ng/mL (< 0.5 ng/mL), and 229 pg/mL (< 18.4 pg/mL), respectively.

**Fig. 2:**
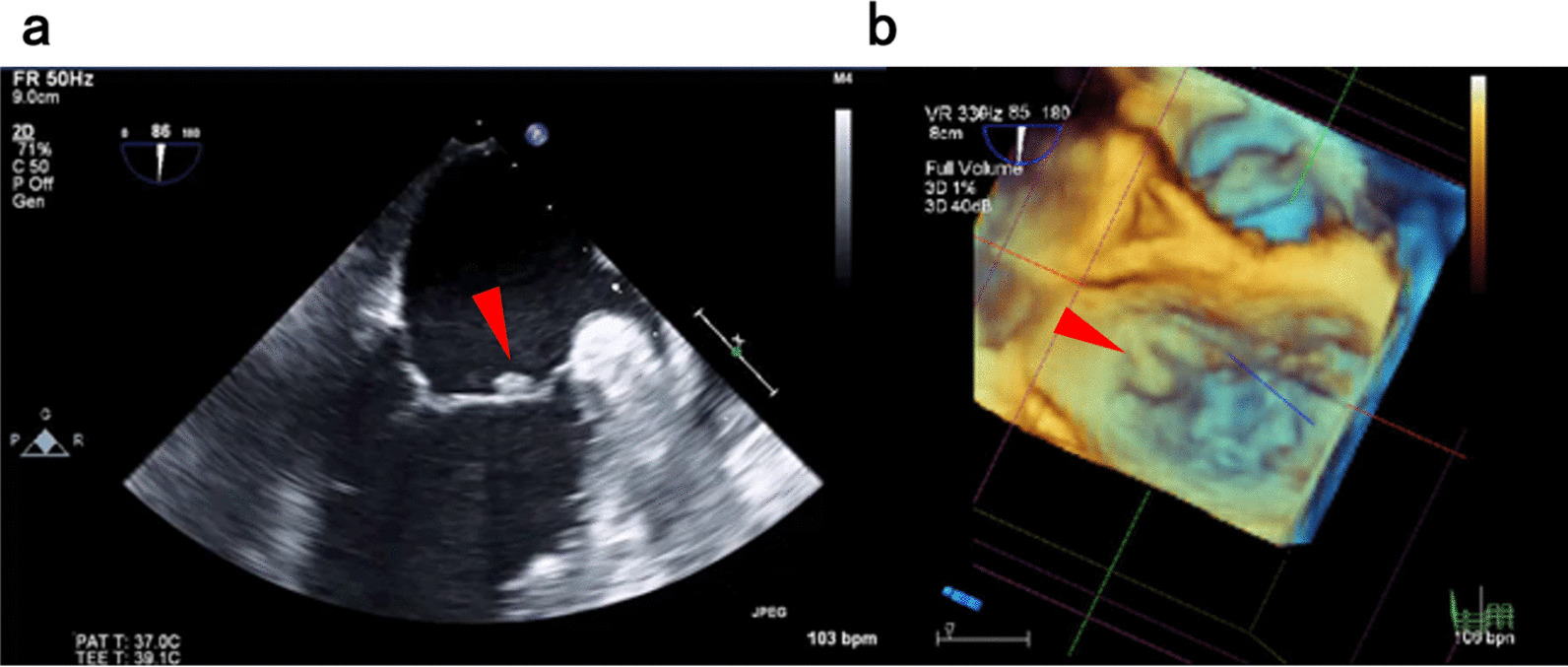
Transesophageal echocardiography images indicate (**a**) vegetation existence (arrowhead) and **b** prolapse of anterior commissure (arrowhead)

Electrocardiography showed sinus tachycardia (heart rate, 110 beats/minute) with no significant arrhythmia or changes in the ST segment. Radiographic examinations, including chest radiography and computed tomography, revealed no significant findings suggestive of infection. Transthoracic echocardiography (TTE) showed left ventricular ejection fraction of 66%, and mild mitral regurgitation was observed. As TTE could not confirm the presence or absence of vegetation because of a poor echo image, transesophageal echocardiography (TEE) was performed. TEE revealed vegetation along the atrial side of the mitral valve and the anterior commissure prolapse of the mitral valve (Fig. 2). There were no findings in the TEE suggestive of a perivalvular abscess.

Blood cultures were drawn, and intravenous ceftriaxone (2 g every 12 hour) was administered. Methicillin-sensitive *Staphylococcus aureus* was subsequently identified in the blood cultures. Magnetic resonance imaging of the brain revealed multiple cerebral infarctions suggestive of septic emboli. After initiation of antibiotic treatment, repeat blood cultures drawn on day 3 were negative.

The patient was discharged with negative blood cultures after 6 weeks of intravenous antibiotic administration. Thereafter, she has been followed up regularly in the outpatient clinic and is progressing well with no recurrence signs.

## Discussion and conclusions

HHT causes vascular abnormalities and, therefore, is considered to be a risk factor for developing cerebral infections. Additionally, numerous recent reports have described the risk of severe extracerebral infections associated with HHT. A retrospective analysis of 353 patients with HHT reported that 47 patients (13.6%) were affected by severe infection [7]. Among them, extracerebral infections accounted for 67% of all infections, and approximately 20% of them were caused by *Staphylococcus aureus*.

Several reports have proposed possible mechanisms of extracerebral infections in patients with HHT. Duval *et al.* [6] hypothesized that severe extracerebral infection complicating HHT is related to bacterial invasion of the nasal mucosa because of cotton packing. Guilhem *et al.* [8] reported that patients with HHT exhibit immunological abnormalities including T CD4, T CD8, and NK cell lymphopenia and increased levels of immunoglobulins G and A.

Despite the high risk of developing severe bacterial infections, infective endocarditis associated with HHT is relatively rare. To our knowledge, there are only eight cases of infective endocarditis in patients with HHT [9–16]. Among them, only two cases were treated medically without valvular surgery (Table 1). Our study is the third case report of infective endocarditis, successfully treated only with antibiotics. In addition to chest auscultation, skin examination is important for early diagnosis of infective endocarditis. Although HHT itself causes erythematous macules on the extremities, these macules are usually painless. Detection of erythematous nodules with tenderness (Osler’s nodes) in this case was the symptomatic key finding that indicated infective endocarditis. Interestingly, Osler’s disease and Osler’s node were named after Sir William Osler. Both are known for their characteristic skin abnormalities; however, in this case, we could properly distinguish them based on whether they were painful. Moreover, early diagnosis based on this skin finding in our patient may have allowed for treatment without surgical intervention.

**Table 1:** Cases of infective endocarditis in patients with hereditary hemorrhagic telangiectasia

Ref.	Age	Sex	Infected valve	Pathogen	Antibiotics	Surgery	Outcome
[9]	62	F	Mitral (prosthetic)	*S. mitis*	Piperacillin + cefazolin	MVR	Survived
[10]	79	F	Mitral	*S. aureus*	Oxacillin	MVR	Dead
[11]	73	M	Aortic (prosthetic)	Unknown	Unknown	AVR	Survived
[12]	69	M	Mitral	*S. aureus*	Oxacillin	–	Survived
[13]	61	F	Aortic (prosthetic)	*S. aureus*	Unknown	AVR	Survived
[14]	65	F	Aortic (prosthetic)	*S. epidermidis*	Unknown	AVR	Survived
[15]	65	M	Pulmonary	*S. epidermidis*	Rifampin + linezolid	Valvuloplasty	Survived
[16]	65	F	Aortic (Prosthetic)	*L. rhamnosus*	Amoxicillin/clavulanate + gentamicin	–	Survived

*S. mitis: Streptococcus mitis*, *S. aureus: Staphylococcus aureus*, *S. epidermidis: Staphylococcus epidermidis*, *L. rhamnosus: Lactobacillus rhamnosus*, *HHT* hereditary hemorrhagic telangiectasia, *F* female, *M* male, *MVR* mitral valve replacement, *AVR* aortic valve replacement

In conclusion, we encountered a rare case of infective endocarditis in a patient with HHT. Recognizing the risk of extracerebral infections, including endocarditis, in patients with HHT is important for early diagnosis and for providing appropriate patient care. This rare case effectively demonstrates the importance of proper diagnosis of skin lesions, which leads to early diagnosis that can prevent progression of infective endocarditis and achieve good clinical course without invasive treatment.

## Data Availability

Not applicable.

## Abbreviations

ACVRL1: Activin type II-like receptor kinase 1
AVM: Arteriovenous malformation
HHT: Hereditary hemorrhagic telangiectasia
TEE: Transesophageal echocardiography
TTE: Transthoracic echocardiography
